# Topological structure and the creation of visual complexity

**DOI:** 10.3758/s13423-026-03008-0

**Published:** 2026-09-15

**Authors:** Ashna M. Shah, Sami R. Yousif

**Affiliations:** 1 Department of Psychology and Neuroscience, University of North Carolina, Chapel Hill, NC USA; 2 Department of Psychology, Ohio State University, 1835 Neil Ave, Columbus, OH 43210 USA

**Keywords:** Topology, Complexity, Spatial cognition

## Abstract

What does it mean for something to be complex? Fundamentally, complexity is a cue that something is less easily represented by our minds. With this fact in view, complexity becomes a tool: By understanding the factors that lead to various impressions of complexity of a given object, we can effectively “reverse engineer” the underlying *form* of the mental representation of that object. Here, we use this tool to explore the ways that topological structure relates to perceived complexity – and, thus, whether, or in what way, topological structure may be part of the representation of objects. In two experiments, we give participants the opportunity to modify simple block-like objects in order to evaluate the topological features that are implicated in their sense of complexity. In a final experiment, using insights gleaned from the previous two, we test specific predictions about topological structure and visual complexity. Altogether, we show that topological structure is clearly implicated in judgments of complexity, including in some key ways that expand existing theories of visual complexity.

## Introduction

**Fig. 1:**
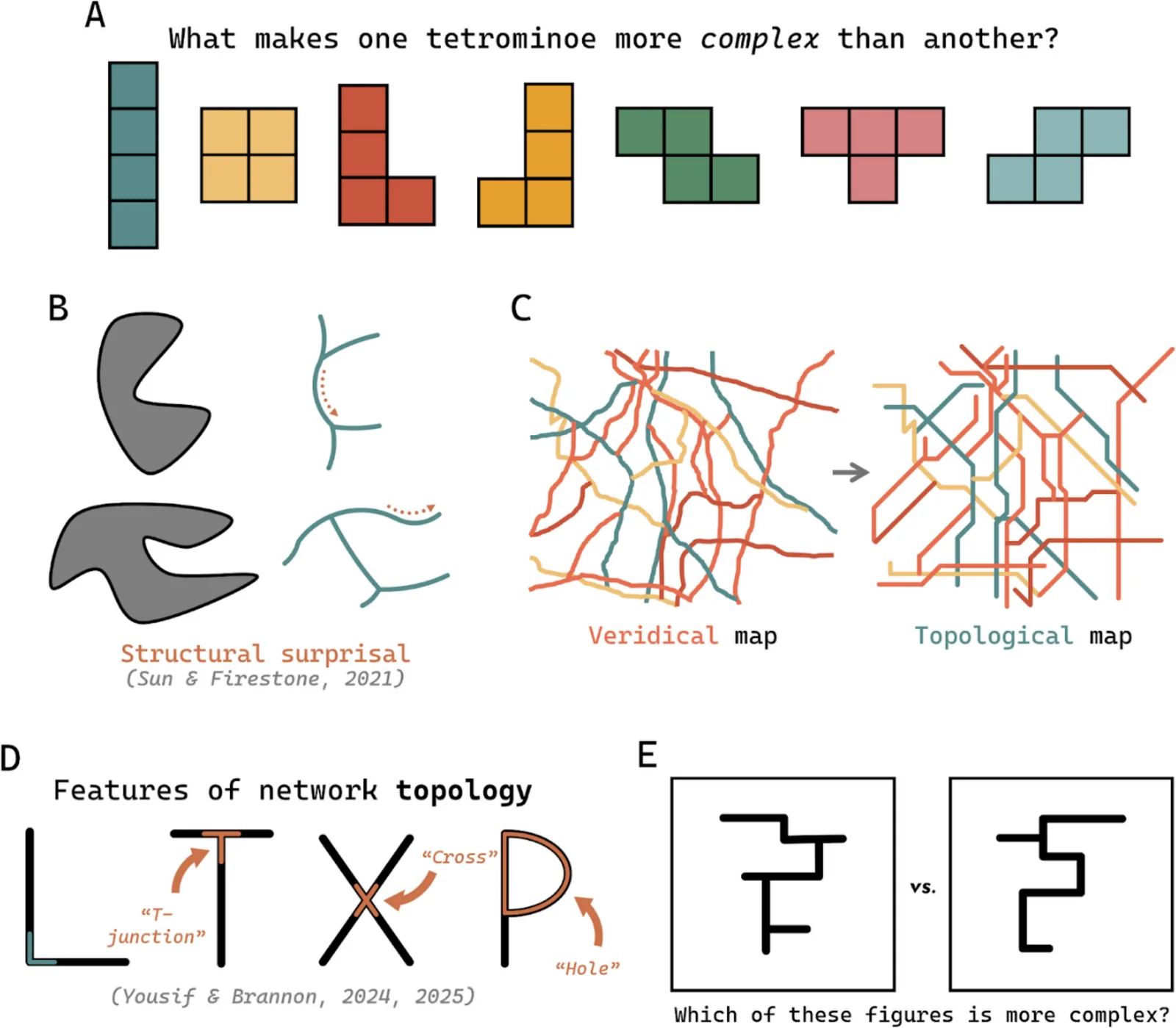
(**A**) Examples of various tetrominoes from the popular game *Tetris*. (**B**) A visual explanation of structural surprisal, per Sun and Firestone (2021). (**C**) An example of a transit system map and its representation as a topological map. (**D**) A visual description of network topology features (Yousif & Brannon, 2024, 2025). (**E**) A comparison of two objects with differing relative complexities

Imagine all the different Tetris pieces (or inspect Fig. 1A). Despite all being made up of exactly four blocks, the various tetrominoes look meaningfully different from one another: The long, 4-by-1 piece (**|**) and the square, 2-by-2 piece are visually “simpler” than the T-piece, for instance. This is to say that the complexity of an object depends not just on the pieces that compose it (i.e., the number of blocks), but also on how precisely those pieces are arranged with respect to one another. What factors govern our sense of what is “simple” and what is “complex”? Here, we take a *topological* approach to answering this question: We offer participants an opportunity to build (and destroy!) objects to make them more or less complex, and we analyze the resulting objects with respect to their topological structure.

All sorts of basic visual features have been implicated in our sense of complexity, ranging from the surface area of an object, to its symmetry along various axes, to the number of turns around its perimeter (see, e.g., Attneave, 1957; Chipman, 1977; Chipman & Mendelson, 1979; Cutting & Garvin, 1987; Day, 1968; Donderi, 2006; Pelli et al., 2006). Other approaches have emphasized image statistics that like self-similarity (Spehar et al., 2016), compactness (Watson, 2011), randomness (Boger et al., 2025; Griffiths & Tenenbaum, 2003), clutter (Oliva et al., 2004; Rosenholtz et al., 2007), and “structural surprise” (Feldman & Singh, 2006; Sun & Firestone, 2021, 2022). But by far the most enduring idea, cross cutting all of these variables, is that complexity is a function of the *compressibility* or *description length* of an entity (see Attneave, 1954; Boger & Firestone, 2026). That is: A simple object is one that can be efficiently described, and a complex object is one that cannot.

Consider for a moment what the complexity of the **|** piece reveals about that object. For one, that object is probably easier to remember than relatively complex objects. It might also be visually easier to detect, at least among its more complex counterparts. This is because the object is of a form that can be easily described, or *compressed* (Attneave, 1954). If describing the object to a friend, you might say, “It consists of four blocks stacked on top of one another.” But how would you describe the T-piece? You would surely have to allocate more words to describing the structure of that object. This is to say that the **|** piece has a shorter *description length* than some of the other tetrominoes, and this computational description length seems to be directly related to our sense of its complexity (Attneave, 1954; see also Forsythe et al., 2011; Machado et al., 2015; Madan et al., 2017; Marin & Leder, 2013; Schlochtermeier et al., 2013). In this way, understanding what makes something simple versus complex is tantamount to understanding the form of the mental representation itself.

Yet even if we understand that complexity is a proxy for understanding mental representations, and even if we know with certainty that *description length* is the right way to understand complexity, a critical question remains unanswered: What is the underlying *language* in which those descriptions are written? If the language represents precise metric properties, for instance, then we might think that changing the angle at which two lines meet (as in the above example) *would* affect its perceived complexity. If the language is instead topological in nature (as we explore here), then such metric changes would matter less, but qualitative changes in topological structure (like the change from an L-shape to a T-shape) would matter more. Thus, by exploring complexity with respect to topological structure, we are essentially asking: Are topological relations part of the *language*, or *format*, of spatial representations of objects (see Yousif, 2022)?

Perhaps the most complete account of judgments of complexity of objects comes from Sun and Firestone (2021), who examined the skeletal structures of shapes in terms of their *structural surprisal* (see Fig. 1B) – defined as the integral of the turning angle of each branch of the skeleton (with a penalty imposed for each additional branch that is added to the skeleton). This sort of measure can straightforwardly explain both why an L-shape is more complex than a |-shape (because it has a greater cumulative turning angle), and why a T-shape is more complex than an L-shape (because it has an additional branch). This metric provides an effective summary statistic for understanding the relative complexity of simple objects and forms. It also leaves open some unanswered questions. For instance, how should the complexity of a hole (as in the loop of an R-shape) be calculated? The hole connects to the main branch of the shape in two different places, so should this branch be penalized twice as an additional branch? Doing so would be counterintuitive, since, in some ways, the addition of holes seems to make an object simpler. An O-shape, for instance, may seem simpler than a U-shape.

### Current study: A topological approach

Here, inspired by recent work showing that adults (see Yousif & Brannon, 2024, 2025) and children (Kittur et al., 2026; Yousif et al., 2025) intuitively understand and perceive the network-topological structure of objects and spaces, we explore how this same sense of topological structure is related to the sense of complexity.

A few things are worth noting about the kind of topological structure studied here. First, representations that emphasize network-topological structure are common in everyday life, as evidenced by the popularity of topological maps for transit systems across the globe (see Fig. 1C). Second, note that this kind of topological representation, which emphasizes *topological relations* (see Fig. 1D), differs from *object topology* (which has been the focus of other work in cognitive science; see Chen, 1982; Kibbe & Leslie, 2016) in several important ways. For instance, from the perspective of object topology, a T-shape and an L-shape are functionally identical; indeed, prior work has treated them as such (see, e.g., Wei et al., 2019). Most work on object topology has focused on only one sort of topological change – the difference between objects with and without holes (see Chen, 1982, 1985, 1990; Kibbe & Leslie, 2016; but see also Lovett & Franconeri, 2017). Third, this approach is not fully mutually exclusive with prior approaches. For instance, approaches that emphasize shape skeletons (see Sun & Firestone, 2021, 2022; see also Ayzenberg & Lourenco, 2019; Ayzenberg et al., 2019) make many of the same predictions that a topological approach would. Yet there are two key virtues of a topological approach:

1. It allows for objects to be described with respect to discrete parts; and
2. There are some predictions that fall out of this approach that may result in some surprising conclusions (see Experiment 3).

Even so, this topological approach is not meant to replace previous approaches, but rather to supplement them.

Are basic topological structures (like T-junctions, crosses, and holes) primitive pieces in a *language* that describe the structure of spatial objects? To investigate people’s sense of complexity through a topological lens, we had participants complete various open-ended block-building (and block-destroying!) tasks. In a first experiment, we asked participants to add blocks to an existing block-based object with the goal of either maximizing or maintaining its original level of complexity. In a second, we asked participants to *remove* blocks from an existing block structure with the goal of either minimizing or maintaining its original level of complexity. In both cases, we analyzed the resulting objects with respect to their topological constituents. In a final experiment, using a speeded-judgment task, we put some of the more surprising predictions of the topological approach (born out of the earlier experiments) to the test.

## Experiment 1: Block-building task

First, we examined how participants selectively added blocks to manipulate visual complexity, whether when instructed to maximize or maintain the original complexity of the object. For us, the critical variable of interest was how the topological structure of the objects changed when participants were asked to maximize versus maintain complexity. The hope was that this approach would give us some preliminary insight into how participants freely created a sense of complexity in an unconstrained task.

### Methods

Pre-registrations for this experiment and subsequent experiments, as well as materials and raw data, are available on our Open Science Framework (OSF) page:

https://osf.io/x4gfb/

#### Participants

Participants were recruited via the Prolific platform. All were adults aged 18 years or older, residing in the USA, and proficient speakers of English. Per our preregistered criteria, we collected data from two separate groups of 50 participants. Participants were excluded if (a) they failed to complete all 24 experimental trials or (b) they demonstrated a clear misunderstanding of the task instructions (e.g., placing blocks not adjacent to existing pieces or forming forbidden 2 × 2 squares). Three participants met these exclusion criteria and were replaced. All participants provided informed consent, and the study protocol was approved by the relevant Institutional Review Board.

#### Stimuli

**Fig. 2:**
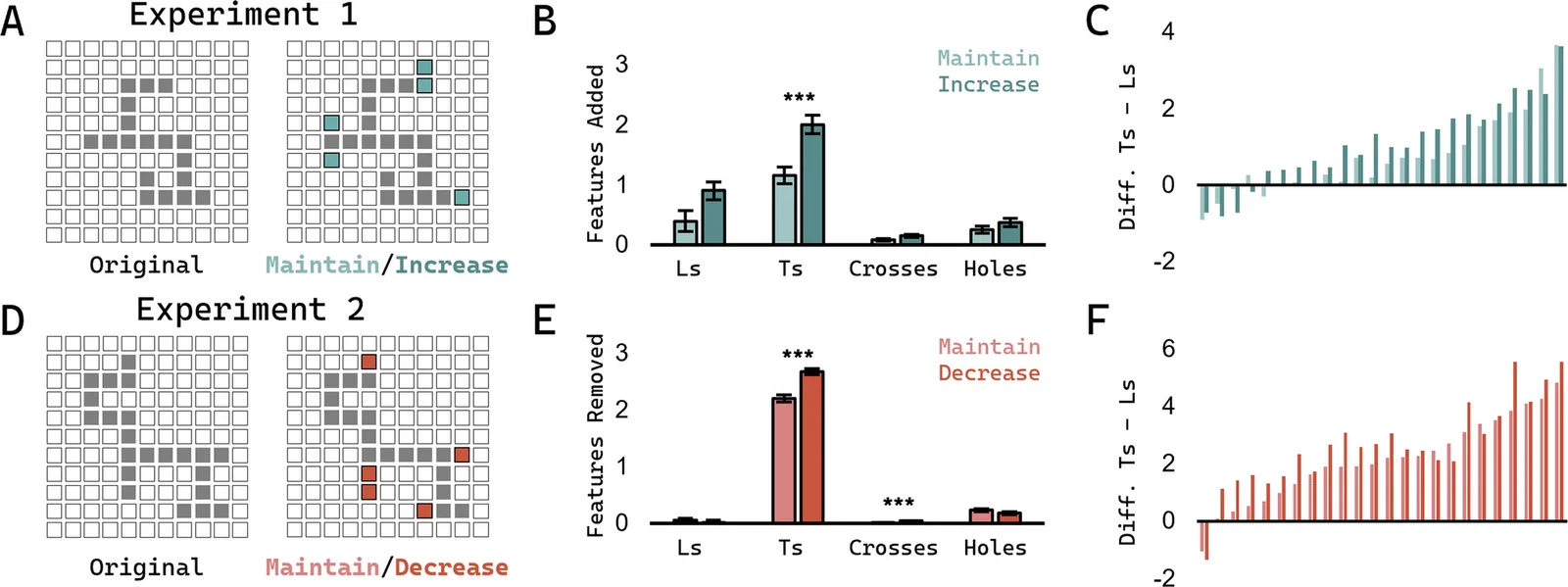
Design and results from Experiments 1 and 2. In the left column (**A**, **D**) are example stimuli showing original objects and their modified counterparts for each experiment’s conditions. The stimuli are displayed in a way to maximize visual clarity in the figure; in reality, all active tiles were blue (see Methods). In the middle column (**B**, **E**) are the average counts of features either added or removed across conditions in the experiments. In the right column (**C**, **F**) are the differences between T-junction and L-junction counts (added in Experiment 1; removed in Experiment 2) for each of the unique 24 stimuli in each experiment. The key pattern here is the fact that the darker bars tend to be higher than the lighter bars, indicating that T-junctions are preferentially added/removed compared to L-junctions

The stimuli consisted of 24 objects created by filling cells in an 11 × 11 grid. Inactive cells were filled white and active cells were filled blue (#0070C0), both with thin black borders. The initial objects occupied as few as 14 cells and as many as 30 cells. No cells along the outer perimeter of the grid were ever initially occupied. Cells activated by the participant were the same blue color, but with a thicker black outline to clearly distinguish original cells from activated cells. A representative example can be seen in Fig. 2A, and the full set of initial objects is available on our OSF page. Across all experiments, the stimuli were block-based objects. These objects have some unique advantages. First and foremost, due to their discrete nature, these objects provide a natural way for us to examine the topological units of interest. Second, and relatedly, their discreteness separates them from prior work, which examines the complexity of more rounded, ambiguous shapes (a difference which may or may not turn out to be substantive). Third, they offer a convenient medium for participants to manipulate the objects in a controlled fashion. Participants have considerable flexibility in how they respond, but the entities they create as a result are still readily analyzable. Finally, as a result of all of the above, these stimuli allow us to study complexity independently from certain low-level properties such as the overall line length or “size” of the objects. Since we can constrain the *number* of blocks that people can add or remove from the display, we ensure that differences in complexity are due to the precise *arrangement* of blocks, rather than their quantity.

#### Task design and procedure

The experiment was administered online via a custom JavaScript interface. On each trial, participants viewed a single unique “Tetris-esque” object rendered on a grid of inactive and active cells. They were instructed to add a fixed number of blocks (by simply clicking on an inactive tile) subject to three rules:

1. Blocks could only be placed on inactive cells immediately adjacent to at least one active block;
2. Original active blocks could not be removed; and
3. No placement could result in a filled 2 × 2 square.

There was no time limit on responses. Participants added five, six, or seven blocks (eight trials each, for a total of 24 trials); there was a counter displayed prominently on the screen indicating how many additional blocks they had left to place. Before the main task, participants completed two practice trials (data not recorded) to familiarize themselves with the interface and rules. The trial order was randomized for each participant.

Critically, one group of participants was asked to maximize the complexity of the object, and another group was asked to maintain its complexity. No formal definition of complexity was provided, allowing participants to rely on their intuitive judgments. A between-subjects design was used to prevent competing goals from influencing participants to adopt mixed approaches across trials. This design also accounts for differing “base rates” of topological features inherent in the items. Because the critical comparisons depend on the *relative* change in each feature across the two conditions, the “base rates” of each feature are effectively controlled (despite each stimulus having a unique number of T-junctions, crosses, and holes).

#### Analysis

The critical factors in our analyses are different topological features, namely L-junctions, T-junctions, crosses, and holes. Once data were collected from all participants, a Python script was run to calculate the total number of L-junctions, T-junctions, and crosses per trial, while holes were scored manually. The count of each topological feature was averaged across participants. We compared the average number of each feature added across conditions. Note that in these analyses, L-junctions, T-junctions, and crosses are not independent. The addition of a cross removes a T-junction; the addition of a T-junction may remove an L-junction. For this reason, it is important that we are not comparing the absolute change in L-junctions and T-junctions, but rather the *relative* change in each feature across two conditions. Thus, we can still assess how instructions related to complexity influence object creation.

### Results and discussion

The results of Experiment 1 can be seen in Fig. 2A–C. As is evident from the figure, participants’ block placements differed systematically by instruction (i.e., whether they were asked to maximize or maintain complexity). In the Increase Complexity condition, participants added a significantly greater number of T-junctions than in the Maintain Complexity condition (*t*(98) = 4.06, *p <*.001, *d* =.81). In contrast, after correction for multiple comparisons, there were no differences between conditions in the counts of L-junctions (*t*(98) = 2.23, *p* =.028, *d* =.45), crosses (*t*(98) = 2.09, *p* =.039, *d* =.42), and holes (*t*(98) = 1.25, *p* =.216, *d* =.25). These patterns are consistent with a topological perspective: We expected that L-junctions would not increase because they are not topologically relevant (they are equivalent to a straight line), while T-junctions would increase because they are topologically relevant (an intersection of two lines). We had predicted that participants would add more crosses in the Increase Complexity condition, but think the lack of difference here may be attributed to the relatively low number of crosses in the dataset overall. There was no specific prediction for holes.

Experiment 1 demonstrates that observers selectively employ topological operations – in particular, adding T-junctions – when instructed to increase the perceived complexity of a shape. The absence of effects for L-junctions, crosses, and holes implies that not all topological transformations contribute equally to intuitive notions of “complexity.”

## Experiment 2: Block-destruction task

In a second experiment, we examined how participants selectively *removed* blocks to manipulate visual complexity, whether to minimize or maintain the complexity of the object. This complementary task allowed us to test whether the same topological features identified in Experiment 1 would be targeted when participants are asked to simplify objects. By comparing the results of addition and removal, we aimed to determine whether participants rely on consistent structural cues to achieve opposing goals.

### Methods

This experiment was identical to the previous experiment except as noted. Two new groups of 50 participants completed the task, one of which was asked to minimize the complexity of the object and one of which was asked to maintain its complexity. No participants were excluded. The stimuli were created in the same manner except that the initial objects occupied as few as 20 cells and as many as 39 cells. A representative example can be seen in Fig. 2D, and the full set of initial objects is available on our OSF page. The procedure was also identical except that participants were instructed to remove a fixed number of blocks subject to two rules:

1. Only existing active blocks could be modified, and
2. Blocks that break the structure into multiple pieces could not be removed. For the analyses, we again compared the average number of topological features across conditions.

### Results and discussion

The results of Experiment 2 can be seen in Fig. 2D–F. Similar to Experiment 1, participants’ block placements differed systematically by instruction. In the Decrease Complexity condition, participants removed a significantly greater number of T-junctions (*t*(98) = 5.47, *p <*.001, *d* = 1.09) and crosses (*t*(98) = 5.13, *p <*.001, *d* = 1.03) than in the Maintain Complexity condition. In contrast, there were no differences between conditions in the counts of L-junctions (*t*(98) = 1.29, *p* =.199, *d* =.26) and holes (*t*(98) = 1.49, *p = *.140, *d* =.30). These patterns are also consistent with a topological perspective: We expected that L-junctions would not decrease because they are not topologically relevant (i.e., they are equivalent to a straight line), while T-junctions and crosses decrease since they are topologically relevant and contribute to judged complexity. There was no specific prediction for holes.

Experiment 2 once again demonstrates that observers selectively employ topological operations – in particular, removing T-junctions and crosses – when instructed to decrease the perceived complexity of a shape.

## Experiment 3: Explicit-judgment task

The first two experiments explored how participants produce complexity, but they did not isolate how features contribute to the perception of complexity. In a third experiment, we examined whether judgments of object complexity are influenced by specific topological features when presented with multiple variants at once. Participants viewed sets of four objects that differed only in the presence of a T-junction and/or a hole and were asked to select either the least or most complex item. This allowed us to directly examine the relative influence of T-junctions and holes on perceived complexity.

### Methods

This experiment was unlike the previous experiments in that participants were not creating or altering objects; instead, they were judging different sets of objects.

#### Preregistration

Our preregistration states that we will ask participants about the items that are most complex. We did not explicitly pre-register our intention to ask about the item that was least complex. We decided to run this extra version after running the first version because we believed it would give us extra insight into participants’ judgments, but we did not pre-register the task again because it was nearly identical to the original task.

#### Participants

Again, two groups of 50 participants each completed the task, one of which evaluated which object in each set was the most complex and one of which evaluated which object in each set was the least complex. Four participants were excluded and replaced for failing to complete all trials.

#### Stimuli

**Fig. 3:**
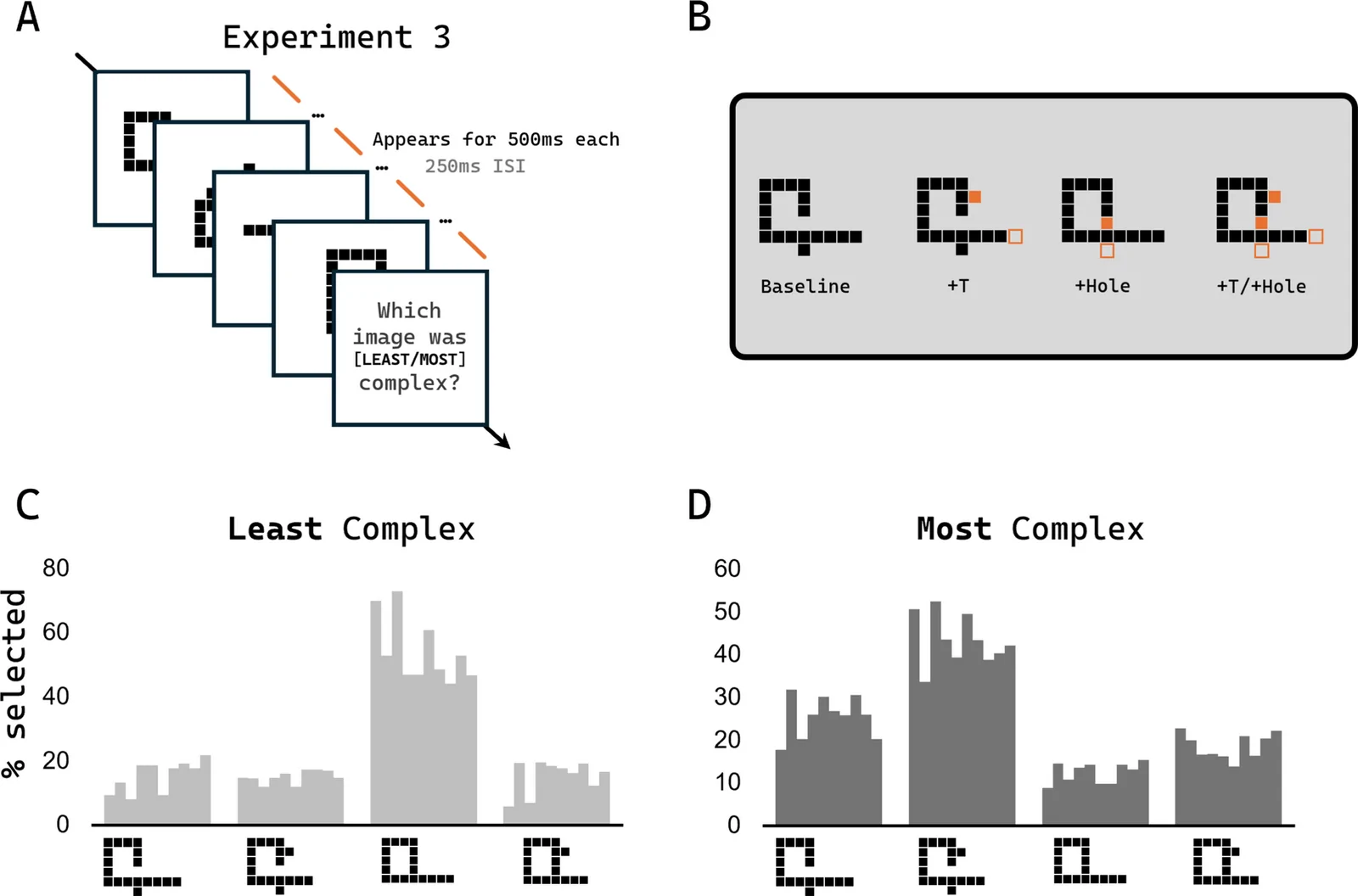
Design and results from Experiment 3. (**A**) Schematic of trial structure showing sequential presentation of four images per trial for 500 ms each, with a 250-ms inter-stimulus interval. Participants indicated which image in each sequence appeared least or most complex by pressing 1, 2, 3, or 4 on the keyboard. (**B**) Labeled examples of each stimulus type (e.g., “Baseline,” “+T,” “+Hole,” “+T/+Hole”) are included for illustration purposes only and were not displayed during the experiment. (**C**) Participant responses showing counts of images selected as the least complex across sets for each object type. (**D**) Participant responses showing counts of images selected as the most complex across sets for each object type. In both (**C**) and (**D**), each bar represents a distinct stimulus set

The stimuli consisted of ten sets of four objects (40 total) created by filling cells in a 12 × 12 grid. Empty cells were transparent with no borders, and filled cells were black with thin white borders. Each set had the following types of objects: a baseline stimulus, the baseline stimulus with one block moved to create a T-junction, the baseline stimulus with one block moved to create a hole, and the baseline stimulus with one block moved to create a T-junction and one block moved to create a hole. The initial objects occupied as few as 18 cells and as many as 33 cells. No cells along the outer perimeter of the grid were ever initially occupied. An example can be seen in Fig. 3B. The full stimulus set is visible on our OSF page.

#### Task design and procedure

The experiment was administered online via a custom JavaScript interface. There were 100 trials total: Each of ten unique sets of four objects was presented ten separate times in random orders and with randomized orientations. On each trial, participants were briefly shown four “Tetris-esque” objects and instructed to indicate which image was the most or least complex. Each object appeared for 500 ms, with a 250-ms inter-stimulus interval. Before the main task, participants completed two practice trials (data not recorded) to familiarize themselves with the interface and rules. The trial order was randomized for each participant.

#### Analysis

The critical factors in our analyses are the type of object chosen across conditions. Permutation tests were used to determine whether participants chose certain feature combinations more often than would be expected by chance.

## Results and discussion

The results of Experiment 3 can be seen in Fig. 3. Similar to Experiments 1 and 2, participants’ judgments were systematically influenced by the presence of topological features. In the Most Complex condition, participants selected the +T objects far more often than would be expected by chance (permutation test; *p <*.001). Additionally, +Hole and +T/+Hole objects were chosen much less often than chance (permutation test; *p <*.001), while there was no difference in how often baseline objects were chosen (permutation test; *p* =.193). In contrast, in the Least Complex condition, participants selected the +Hole object far more often than would be expected by chance (permutation test; *p <*.001), while baseline, +T, and +T/+Hole objects were chosen much less often than expected by chance (permutation test; *p <*.001).

Experiment 3 demonstrates that holes have a strong negative effect on people’s judgments of complexity. While items with T-junctions were chosen as more complex than otherwise equivalent items, those with holes were judged as less complex than otherwise equivalent items. Furthermore, the +T/+Hole object was chosen as being more complex than the +Hole object, but less complex than the baseline and +T objects, implying that holes may have a stronger effect on judged complexity than T-junctions.

These results may seem to be in tension with the prior experiments, in which we did not observe an effect of holes. However, what we found here is that the addition of holes *decreased* perceived complexity – a pattern neither of the previous experiments could have revealed. In the first experiment, participants were tasked with increasing or maintaining complexity; if holes reduce complexity, it makes sense that they would not have added many. In the second experiment, participants were tasked with decreasing complexity, but they could only remove blocks; they were not able to create holes. In this way, the results across experiments are congruent.

## General discussion

Everywhere we look, we are struck by a sense of *complexity*. Complexity guides how we interact with the world – what things we notice and remember (Boger & Keil, 2024), what things we are curious about (Sun & Firestone, 2021), what things we find beautiful (Forsythe et al., 2011; Spehar et al., 2016; Van Geert & Wagemans, 2020), and even how we communicate (Sun & Firestone, 2022). Yet what it means precisely for anything to be *simple* versus *complex* remains mysterious, despite strong theories about representations of complexity. To wit: Even once we understand that complexity can be operationalized with respect to an item’s *description length*, we must understand what *language* that description is written in.

Here, we have studied complexity with respect to a particular representational form: *topological relations*, or *network topology*. We have shown that complexity can be understood in part with respect to topological structure – that certain “words” in this spatial “language” are systematically related to perceived complexity. In Experiments 1 and 2, we showed that people systematically add T-junctions and crosses to objects when asked to increase their complexity but systematically remove those same features when asked to reduce their complexity. In a final experiment, we showed that holes also have a unique (and possibly counterintuitive) relationship with complexity, such that the addition of holes robustly decreases the perceived complexity of an object. While some of these results are consistent with prior work (see especially Sun & Firestone, 2021), others would not be straightforwardly explained by existing theories of complexity.

Ultimately, though, our question is not about complexity per se but about the underlying form of object representations that causes something to be perceived as more or less complex in the first place. Is there, for instance, a “geometric language of thought” underlying these object representations (see Sablé-Meyer et al., 2021, 2022; see also Al Roumi et al., 2021; Amalric et al., 2017; Yousif & Epstein, 2026)? There is one reason we believe that the present results may speak directly to the representation format, or “language of thought,” underlying spatial representation: The fact that the *addition* of a hole *reduces* perceived complexity. Such a finding is analogous to numerical illusions in which the addition of information into a display results in a decreased impression of number (see Franconeri et al., 2009; Waterhouse & Yousif, 2025). Cases like these are revealing because they cannot be explained by any sort of “more is more” bias, and, in that way, almost certainly reveal something about the underlying units of representation. Thus, even if one is not convinced that object representations are ultimately represented by topological building blocks, they might still take seriously the counterintuitive relationship between holes and perceived complexity.

Why does the addition of a hole reduce complexity? If we think that holes, like other topological building blocks, act as “words” in a representational language, then it is easy to see how the formation of such a word reduces description length. This is no different from how a sequence of numbers might be easier to remember if it resembles famous dates (e.g., the sequence 121517891989 is easier to remember when associated with three historically significant events), or how a specific position on a chessboard might be easier to remember if it is assigned a specific name (e.g., “Ruy Lopez, Morphy Defense”). Holes provide a nameable part while functionally reducing the number of open paths in a figure (unlike the addition of T-junctions or crosses, which, unless also part of an additional hole, always necessarily entail the addition of a new path). In this analogy, adding a T-junction or a cross might be viewed as equivalent to adding a new “branch” to the sequence (e.g., 198…), whereas adding a hole might be viewed as equivalent to adding one more digit to create a more memorable date (i.e., 1989). Both are functional units, but one increases complexity while the other decreases it. Thus, according to this view, the reduction in complexity following the addition of holes provides particularly strong evidence that holes are a functional part of the mental representation since such an effect cannot be explained by any sort of “more is more” bias (see also Yousif et al., 2026).

Another possibility is that the reduction in complexity is somehow related to the perceived physical stability of the object. The connection of various branches to form a hole functionally reduces the “degrees of freedom” an object has to move. In many of our stimuli, the addition of a hole leaves only one “flexible” branch to the object (whereas without that hole, it would have several). Perhaps perceived complexity is somehow related to the perceived changeability of any object.

Although these findings meaningfully constrain theorizing about visual complexity, we note that the findings here do not, and could not, constitute a complete theory of visual complexity. Instead, our suggestion is that this is a fruitful lens through which to understand it. These findings build on the work of Sun and Firestone (2021) by highlighting certain cases in which the addition of branches does not straightforwardly increase complexity. That is: In their formulation, every unique branch increases complexity. Here, in contrast, we show that additional branches may *decrease* complexity – if the additional branch results in the creation of a hole. Additionally, the topological approach offers an account of complexity that may encompass a more general class of stimuli (e.g., things like topological maps). Even so, it is not yet clear how the mind would combine topological building blocks to create more expansive structures. It remains unclear whether this form of representation is computationally plausible for more intricate objects and spaces. Future work may address such questions.

## Conclusion

We have shown that certain simple topological building blocks have a systematic relationship with complexity, such that, for instance, the addition of T-junctions and crosses results in an increased sense of complexity, whereas the addition of holes results in a decreased sense of complexity. These findings provide clues as to how objects may be represented in the first place — namely, with respect to an underlying mental representation of topological structure.

## Data Availability

Data and materials are available at: https://osf.io/x4gfb/
